# Effects of Mobile Phone Use on Gait and Balance Control in Young Adults: A Hip–Ankle Strategy

**DOI:** 10.3390/bioengineering10060665

**Published:** 2023-06-01

**Authors:** Zijun Lu, Xinxin Zhang, Chuangui Mao, Tao Liu, Xinglu Li, Wenfei Zhu, Chao Wang, Yuliang Sun

**Affiliations:** Department of Exercise Science, School of Physical Education, Faculty of Sports and Human Sciences, Shaanxi Normal University, Xi’an 710119, China; luzijun@snnu.edu.cn (Z.L.); zhangxx9606@snnu.edu.cn (X.Z.); maocg117@snnu.edu.cn (C.M.); liutao0604@snnu.edu.cn (T.L.); lixinglu@snnu.edu.cn (X.L.); wzhu@snnu.edu.cn (W.Z.)

**Keywords:** dual-task, gait alteration, motor control, mobile phone use, young adults

## Abstract

Background: This study aimed to derive the effects of walking while using a mobile phone on balance perturbation and joint movement among young adults. Methods: Sixteen healthy college students with no history of brain injury were tested. The participants were asked to walk under four different conditions: (1) walking, (2) browsing, (3) dialing, and (4) texting. Indicators related to balance control and lower limb kinematic/kinetic parameters were analyzed using the continuous relative phase and statistical nonparametric mapping methods. Results: Walking while using a mobile phone slowed participants’ gait speed and reduced the cadence, stride length, and step length. The posterior tilt angle (0–14%, 57–99%), torque of the hip flexion (0–15%, 30–35%, 75–100%), and angle of the hip flexion (0–28%, 44–100%) decreased significantly. The activation of biceps femoris and gastrocnemius, hip stiffness, and ankle stiffness increased significantly. This impact on gait significantly differed among three dual tasks: texting > browsing > dialing. Conclusion: Che overlap of walking and mobile phone use affects the gait significantly. The “hip–ankle strategy” may result in a “smooth” but slower gait, while this strategy was deliberate and tense. In addition, this adjustment also increases the stiffness of the hip and ankle, increasing the risk of fatigue. Findings regarding this effect may prove that even for young healthy adults, walking with mobile phone use induces measurable adjustment of the motor pattern. These results suggest the importance of simplifying the control of the movement.

## 1. Introduction

Human motor tasks such as standing, level walking, and stair negotiation utilize attention [1]. Individuals often multitask daily, including walking while talking with others or using mobile phones. Multitasking involves divided attention and consequently affects performance, indicating a strong association between cognitive load and motor control [2,3,4].

Walking while using mobile phones has become indispensable to people’s daily lives. In the “State of Mobile 2022” report, App Annie shows that people spent more time than ever before in mobile apps, reaching 4.8 h per day in the top mobile-first markets [5]. Several studies found that compared to other groups, the young spend more time on mobile phones [6], especially college students [7]. An immersive virtual experiment found that college students’ attention would be distracted when crossing the road while using mobile phones, missing crucial visual information in the streets, which increases the danger [8]. Another study found that walking up/down a hill while using mobile phones would increase the chance of falling by 83% [9]. Therefore, the effect of walking while using mobile phones on gait is so essential that it deserves further study.

Walking while using a mobile phone is a typical dual task, which could affect how we walk and perceive our surroundings. This is evidenced by a decrease in step speed and stride length, a longer double support time, an increase in the shift of COG (Center of Gravity), etc. [9,10,11]. These studies’ mobile phone use tasks differed, leading to different results. Using mobile phones to send messages in different ways (e-mail, message apps, editing, etc.) was the most frequently tested task [10,12,13,14], followed by talking [9,15,16], reading [15,17], and surfing the internet [18]. Nevertheless, only a few studies compared the impacts on gait among different tasks [19].

Age and environment are also investigated parallelly to reveal the influence on mobile phone gait. Compared with young adults, the influences of using a mobile during walking are more significant in the elderly [13]. Additionally, the natural environment occupies more cognition than the lab environment while walking [9,13]. In recent years, studies focusing on the gait of tasks closest to daily life have increased, such as ascending and descending the staircase [9]. Several studies investigated the effect of sex on this influence, but there was no significant difference between the groups [17].

However, the previous studies mainly analyzed the difference in joint movement and spatial–temporal parameters, needing more stability investigation. Therefore, to reveal the underlying mechanism of motor control patterns induced by mobile phone tasks during level walking, a quantitative measure of stability and electromyography (EMG) are needed. With this in mind, this study compared the changes in lower limb gait among walking, browsing, dialing, and texting. The aim was to derive the effects of walking while using a mobile phone on balance perturbation and joint movement among young adults. Based on previous studies, we hypothesized that (1) using a mobile phone may decrease the gait speed, hip flexion, and ankle plantar flexion; (2) the range of body movement in the sagittal plane may decrease; (3) this impact on gait was significantly different among three dual tasks: texting > browsing > dialing.

## 2. Materials and Methods

### 2.1. Participants

Based on this hypothesis, 16 healthy young participants with no history of brain injury or other neurological conditions were tested (8 males, 8 females; age = 23.4 ± 1.32 years; height = 169.56 ± 8.70 cm; mass = 59.75 ± 10.31 kg, mean ± SD). The sample size was calculated using G-Power software (Ver.3.1) with I error rate ≤ 0.05, statistical power ≥ 0.99, and anticipated Cohen D effect size ≥ 0.8. The inclusion criteria were as follows: all possess and regularly use a web-enabled mobile phone (in a daily situation). Written informed consent was obtained from all participants before testing.

### 2.2. Apparatus and Measurement

The walkway (8 × 1.5 m) had one 900 × 600 mm force platform (Model 9260AA6, Kistler Instrument, Basel, Switzerland). The walkway was situated in a volume covered by an optoelectronic movement analysis system comprising 10 infrared cameras (Oqus700+, Qualisys AB, Göteborg, Sweden). Electromyography (EMG) was measured using Delsys (DELSYS Trigno TM Wireless Biofeedback System, Middlesex, MA, USA).

**Figure 1:**
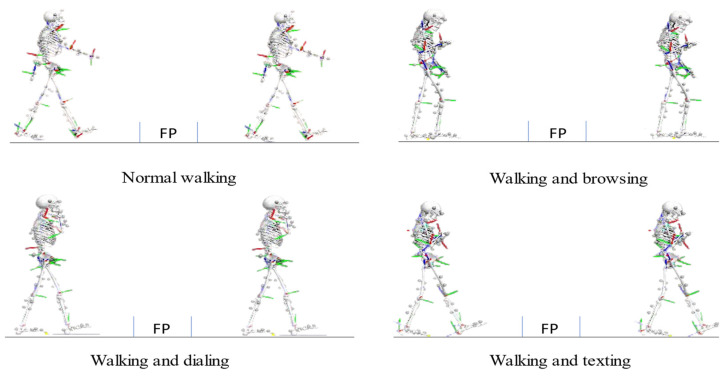
Four conditions were compared: normal walking, walking and browsing, walking and dialing, and walking and texting.

Before testing, the laboratory temperature was adjusted to 27 °C to avoid temperature interference with the subjects’ gait. Each participant’s skin was prepared by shaving the body hair and cleaning the skin with alcohol to reduce impedance. After the warm-up via stair-stepping (step-ups on a 20 cm step, 2 min, self-selected cadence), the subjects were required to test the maximum voluntary contraction (MVC), including gluteus medius (GM), rectus femoris (RF), vatsus lateralis (VL), biceps femoris (BF), gastrocnemius (GAS), and soleus (SOL) of the left lower limb. The participants were asked to walk on a dry 8 m walkway at a self-selected spontaneous speed (which had been tested before) under four different conditions: (1) normal walking without other tasks; (2) walk while texting; (3) walk while dialing; (4) walk while browsing the web. Each condition was performed 3 times in a randomized order (to control the practice effects). All investigators were thoroughly trained, and all investigations were carried out under constant conditions (Figure 1). To ensure that the kinetic data were synchronized with mobile phones, the subjects were required to use the mobile phones continuously during the test.

For the texting condition, one of the investigators sent a message to the participant from another room, informing them to keep typing to answer a question during the walking. For the dialing condition, an investigator called the participant from another room. After he/she answered the call, common knowledge sports questions had to be answered. The investigator asked a new question if the participant could not answer this question. Questions covered the topics of football, basketball, volleyball, table tennis ball, etc. (e.g., “Who is your favorite basketball player? Why is this player your favorite?”). The browsing condition included browsing the website carefully and answering the question about the website after walking. All participants typed the message using both hands while performing walking with texting and freely held the mobile phone in their dominant hand when walking while dialing and browsing the web. During the test, the laboratory was required to be quiet to avoid noise disturbance to the subjects’ gait.

### 2.3. Kinematic and Kinetic Data Reduction

This research intercepted a complete gait cycle, starting with the left heel’s first strike (on the force platform) and ending with its second strike (on the ground). All angles selected were formed with the rotation of distal segments concerning proximal segments. This research divides a gait cycle into 4 phases: double stance phase-I, single stance phase, double stance phase II, and swing phase.

The relevant kinematic parameters are determined as follows. Gait events of the foot-strike and foot-off were identified with the Visual 3D Software using a threshold of 20 N on the vertical ground reaction force. Whole body COM position data were calculated using a 13-segment model with the weighted sum method. The center of pressure (COP) position in the AP and ML directions was calculated using the ground re-action forces/torques measured with the force plates at a sampling rate of 1000 Hz.

All kinematic signals were filtered through a fourth-order Butterworth digital filter at cut off frequencies of 14 Hz [20].

**Figure 2:**
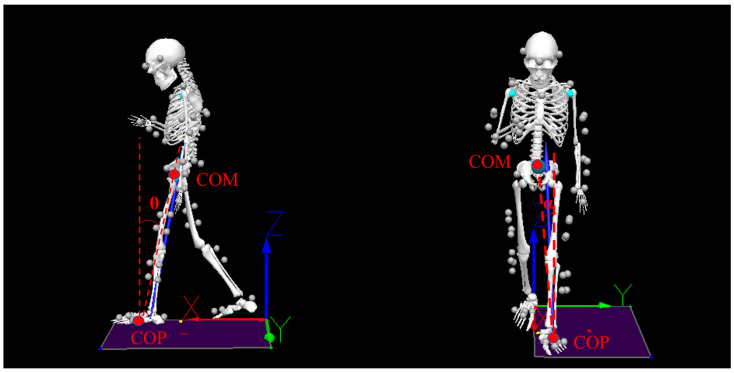
Diagrammatic illustration of COM-COP inclination angle in the sagittal (θ) and frontal (α) plane (under texting condition).

The COM-COP IA is defined as follows: the angle formed by the interaction of the line connecting the COP and COM with a vertical line through the COP, which was calculated for each frame using the horizontal COM-COP separation distance and the corresponding vertical COM height [21] (Figure 2).

COM−COP IAX=ATAN(COGX−COPXCOGZ−COPZ)∗180/PI( )

COM−COP IAY=ATAN(COGY−COPXCOGY−COPZ)∗180/PI( )

After calculating the average number of the subjects’ steps, the stride-to-stride variability was assessed with the coefficient of variation (CV) of the stride time (ST), defined as follows [22]:

CV of stride time (%)=standard deviation (ST)mean (ST)×100

All kinetic signals were recorded at a sampling rate of 1000 Hz and filtered through a fourth-order Butterworth digital filter at cutoff frequencies of 100 Hz [21]. This research used joint torque of the hip, knee, and ankle as kinetic indicators. All data were standardized with the described formula below:

SD=Joint momentHeight×Weight

where *SD* indicates the data after standardization.

$$\mathrm{Joint}\;\mathrm{stiffness}=\frac{\Delta M}{\Delta \theta }$$

where ∆M means the change of joint torque during stance, ∆θ means the change of joint angle during stance.

All EMG signals were recorded at a sample rate of 2000 Hz and filtered with a zero phase fourth-order Butterworth filter, with a band-pass of 20–500 Hz after removing the mean value. This research analyzed the left lower limb’s root mean square (RMS) and integral electromyographic (iEMG) of GM, RF, VL, BF, GAS, and SOL. After being filtered, the data were normalized by dividing it with the MVC data, which has been intercepted (keep stable for 3 s) for an average.

$$\mathrm{RMS}=\sqrt{\frac{\sum_{i=0}^{N}\mathrm{Data}{\left[i\right]}^{2}}{N}}\;\mathrm{iEMG}=\sum_{i=0}^{N}\left|\mathrm{Data}\left[i\right]\right|\times \Delta t$$

### 2.4. Statistical Analysis

Statistical analyses were performed using SPSS (SPSS Statistics v 26, IBM Corp., Armonk, NY, USA) and MATLAB (MATLAB R2016b, The MatWorks Inc., Beltsville, MD, USA). One-way repeated-measures ANOVA implemented in the spm1d toolbox was conducted to analyze the effect of 4 different mobile phone tasks on gait, except for the spatial–temporal parameters, EMG, and joint stiffness variables as they are scalar values. For comparison of values, data distribution was analyzed for normality. An unpaired *t*-test tested differences between the sexes in each task and gait parameter. As there were no differences in gait parameters between the sexes, a one-way repeated-measures ANOVA with a within-subject factor of four tasks (walking, browsing, dialing, and texting) was conducted to examine the effect of mobile phone use on gait alterations. If ANOVA indicated a significant interaction, Tukey’s HSD test was used for multiple comparisons. To account for multiple comparisons, *p*-values were adjusted using the False Discovery Rate (FDR) method with q = 0.05 and c(V) =1. Statistical significance was assumed when *p* < 0.05. When significant differences were observed, Cohen’s measures of effect size (ES) were computed. For spm1d condition, those measures of effect size were computed for each time point and averaged for the duration of the significant clusters [13].

## 3. Results

### 3.1. Spatial–Temporal Parameters

**Figure 3:**
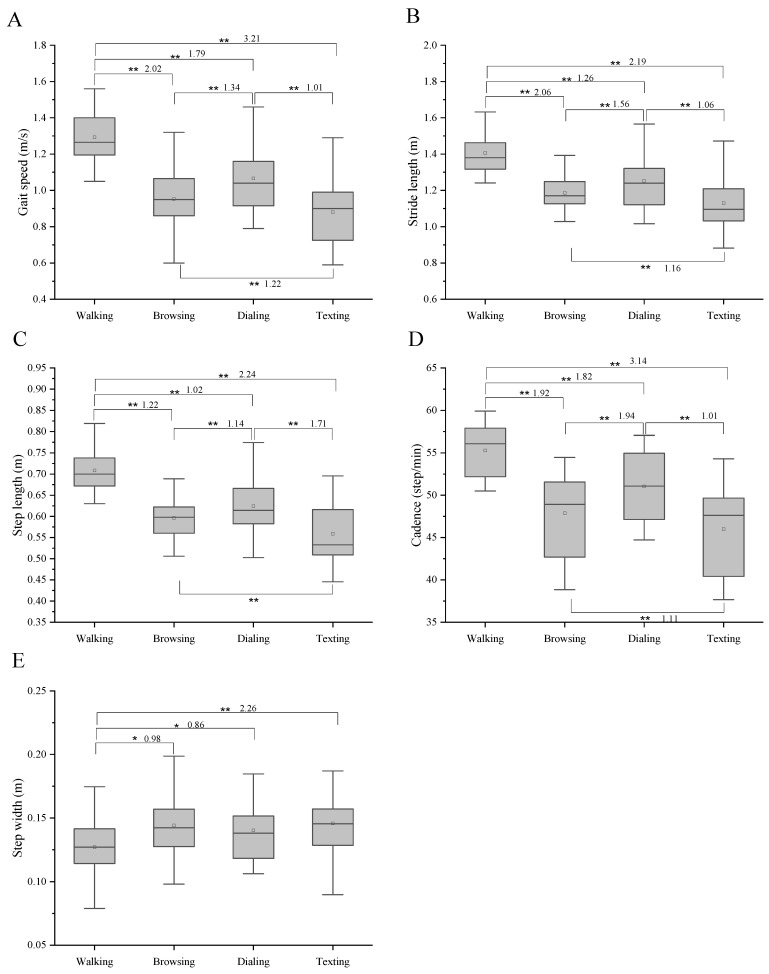
Results of two-way repeated-measures ANOVA and multiple comparisons for each gait parameter (*n* = 16). * Significant difference between the two tasks. (*p* < 0.05). ** Significant difference between the two tasks (*p* < 0.01). Effect size was shown after the * (**). (**A**) represents the gait speed. (**B**) represents the stride length. (**C**) represents the step length. (**D**) represents the cadence. (**E**) represents the step width.

**Figure 4:**
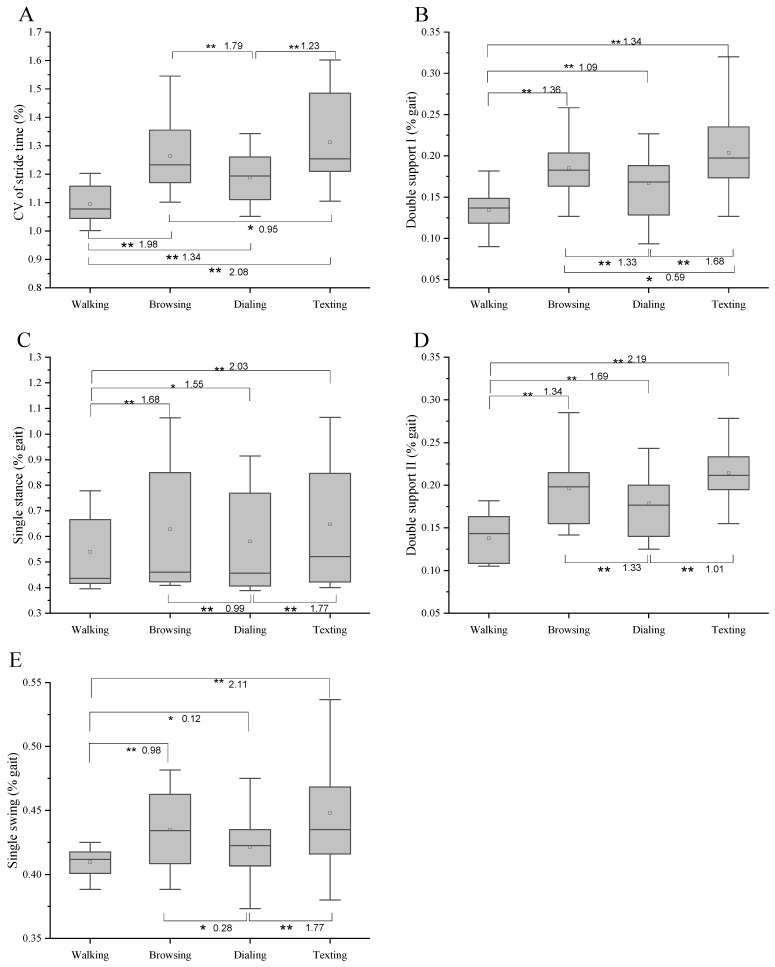
Results of one-way repeated-measures ANOVA and multiple comparisons for each time parameter (*n* = 16). * Significant difference between the two tasks. (*p* < 0.05). ** Significant difference between the two tasks (*p* < 0.01). Effect size was shown after the * (**). (**A**) represents the CV of stride time. (**B**) represents the double support I phase. (**C**) represents the single stance phase. (**D**) represents the double support phase II. (**E**) represents the single swing phase.

Walking while texting, dialing, and browsing the web all slowed participants’ gait speed, reducing their cadence, stride length, and step length (Figure 3). In contrast, the CV of stride time increased, and the time of single support and single stance increased significantly too (Figure 4). All the mentioned differences among walking and three dual tasks are significant (*p* < 0.05, *p* < 0.01).

Figure 3 and Figure 4 show the results of the one-way repeated-measures ANOVA and multiple comparisons for each spatial–temporal parameter. Significant differences among four conditions were observed in gait speed, stride length, step length, cadence, CV of stride time, and double support I (*p* < 0.01). However, step width was the only difference observed among walking and the other three conditions. There was no significant difference between browsing and texting for the time of single stance, double support II, and single swing.

### 3.2. Kinematic, Kinetic, and EMG

**Figure 5:**
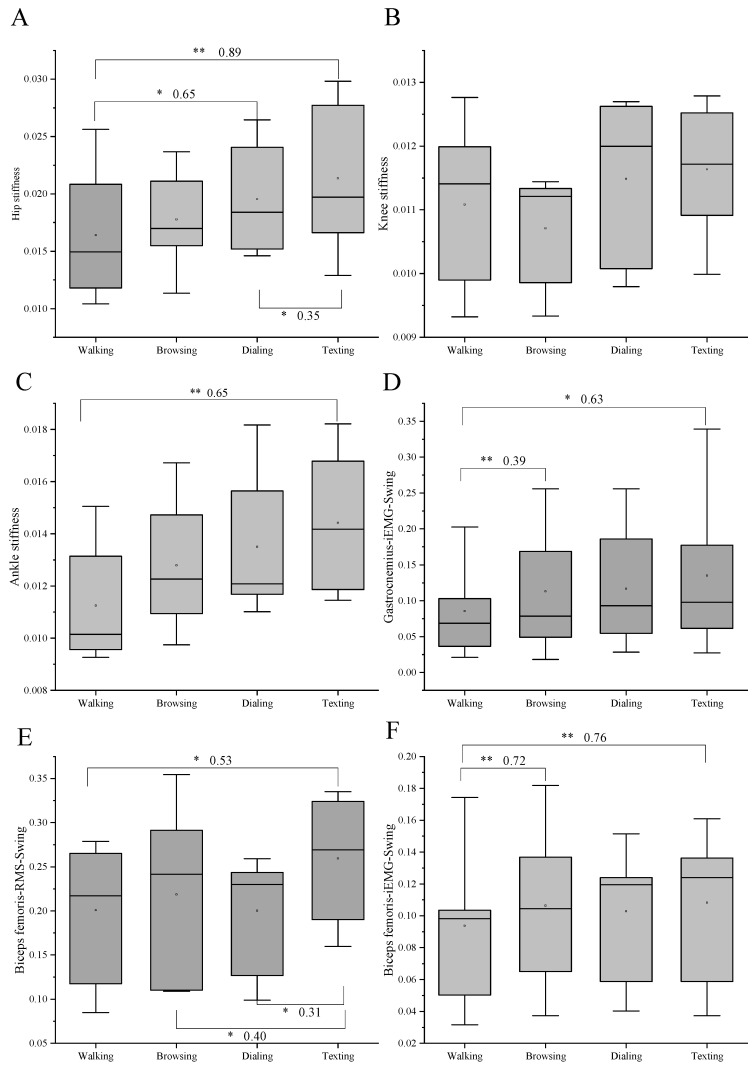
Results of one-way repeated-measures ANOVA and multiple comparisons for EMG and joint stiffness (*n* = 16). * Significant difference between the two tasks. (*p* < 0.05). ** Significant difference between the two tasks (*p* < 0.01). Effect size was shown after the * (**). (**A**) represents the hip stiffness. (**B**) represents the knee stiffness. (**C**) represents the ankle stiffness. (**D**) represents the iEMG of gastrocnemius during swing phase. (**E**) represents the RMS of biceps femoris during swing phase. (**F**) represents the iEMG of biceps femoris during swing phase.

Figure 5 shows the difference in joint stiffness and EMG among the four conditions. Compared to walking, the hip stiffness increased in the texting and dialing condition (*p* = 0.015, ES = 0.65; *p* < 0.01, ES = 0.89). There was also a significant difference between dialing and texting (*p* = 0.043, ES = 0.35). The ankle stiffness also increased more greatly under the texting condition than the walking condition (*p* = 0.022, ES = 0.65). The iEMG of gastrocnemius during the swing phase increased in texting and browsing condition compared to the walking condition (*p* = 0.017, ES = 0.63; *p* < 0.01, ES = 0.39). The RMS of biceps femoris during the swing phase decreased in walking, browsing, and dialing conditions compared to the texting condition (*p* = 0.021, ES = 0.53; *p* = 0.016, ES = 0.40; *p* = 0.041, ES = 0.31). The iEMG of biceps femoris during the swing phase increased in browsing and texting conditions compared to the walking condition (*p* < 0.01, ES = 0.72; *p* < 0.01, ES = 0.76).

**Figure 6:**
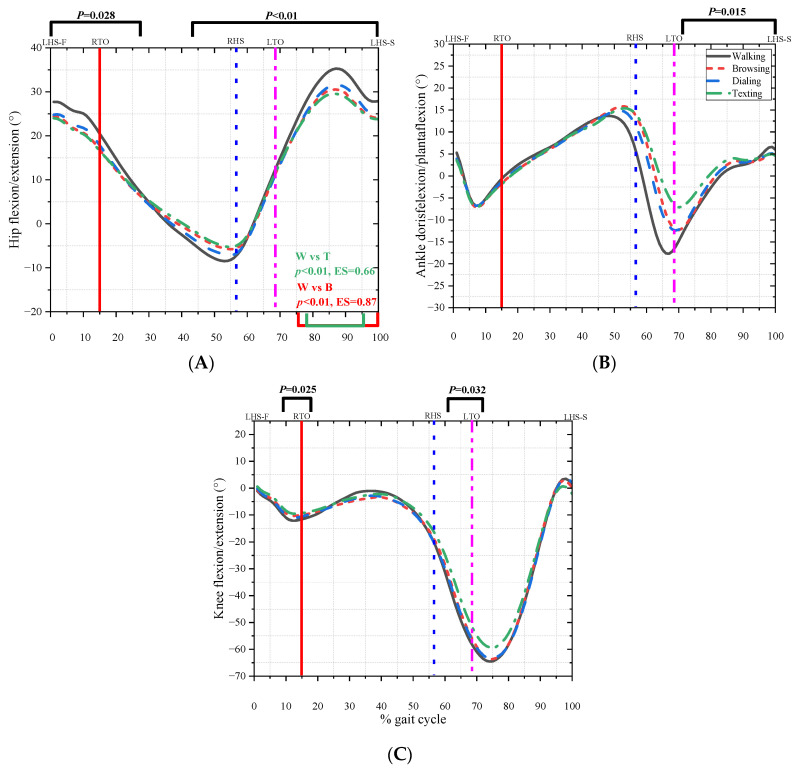
The kinematic parameters in four conditions. (**A**) represents the angle of hip flexion and extension. (**B**) represents the angle of ankle doris flexion and plantar flexion. (**C**) represents the angle of knee flexion and extension.

Walking while texting, dialing, and browsing significantly affects gait patterns, which increases the risk of balance perturbation and lower limb injury. Figure 6 shows the difference in kinematic parameters among the four conditions. The angle of hip flexion decreased during the first double stance phase (0–28%, *p* = 0.028), second double stance phase, and swing phase (44–100%, *p* < 0.01). Normal walk was significantly different from browsing (75–100%, *p* < 0.01, ES = 0.87) and texting (78–95%, *p* < 0.01, ES = 0.66). The angle of knee flexion decreased (9–16%, *p* = 0.025; 67–72%, *p* = 0.032) significantly, and the angle of the ankle plantar flexion decreased during the swing phase (74–100%, *p* = 0.015).

**Figure 7:**
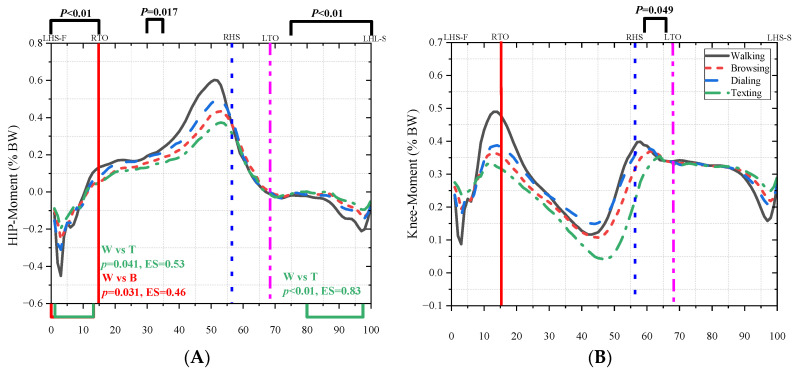

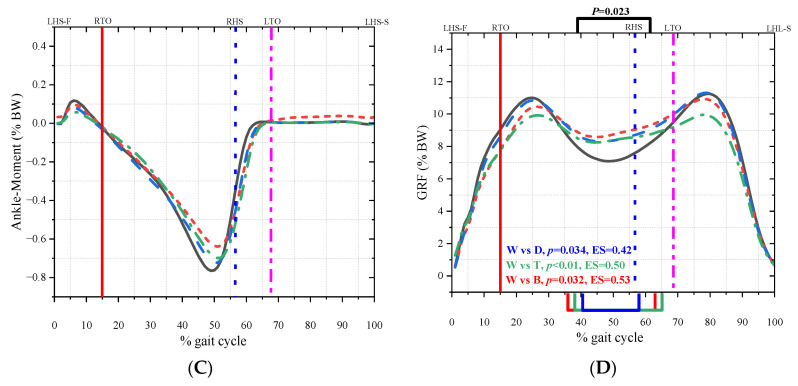
The kinetic parameters in four conditions. (**A**) represents the torque of hip flexion and extension. (**B**) represents the torque of knee flexion and extension. (**C**) represents the torque of ankle doris flexion and plantar flexion. (**D**) represents the trend of vertical Ground reaction force.

Figure 7 showed the difference in kinetic parameters among the four conditions. The torque of the hip flexion significantly decreased during the first double stance phase (0–15%, *p* < 0.01), the middle phase of the single stance phase (30–35%, *p* = 0.017), and the swing phase (75–100%, *p* < 0.01). Normal walk was significantly different from browsing (0–14%, *p* = 0.031, ES = 0.46) and texting (2–14%, *p* = 0.041, ES = 0.53; 80–97%, *p* < 0.01, ES = 0.83). There was also a difference in the torque of the knee extension during the second double stance phase (64–68%, *p* = 0.049). The GRF increased during the single stance phase and initial second double stance phase (38–63%, *p* = 0.023). The normal walk was significantly different from dialing (40–58%, *p* = 0.034, ES = 0.42), texting (38–65%, *p* < 0.01, ES = 0.50), and browsing (36–58%, *p* = 0.032, ES = 0.53).

**Figure 8:**
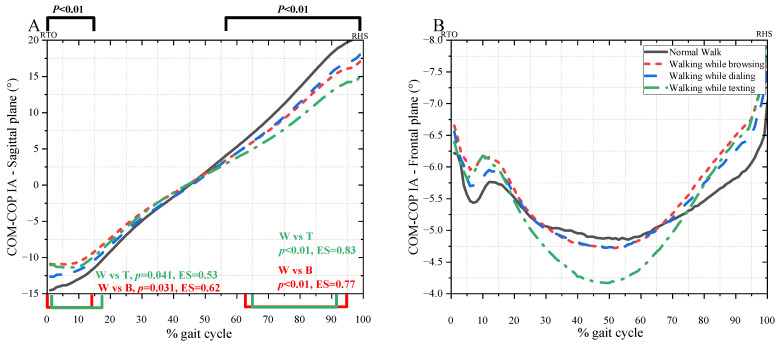
The COM-COP IAs in the sagittal plane (**A**) and frontal plane (**B**).

The instantaneous COM-COP IAs in the sagittal plane and frontal plane were illustrated in Figure 8. The COM-COP IA in the sagittal plane decreased with mobile phone use during the single stance phase (0–14%, 57–99%, *p* < 0.01) (Figure 8A). Normal walk was significantly different from browsing (0–14%, *p* = 0.041, ES = 0.06; 62–95%, *p* < 0.01, ES = 0.77) and texting (2–17%, *p* = 0.041, ES = 0.53; 65–92%, *p* < 0.01, ES = 0.83), respectively.

## 4. Discussion

In this study, we measured biomechanical indicators of gait performance to assess the difference of dual ecological tasks on spatial–temporal parameters, joint angle, joint torque (post-standardization), COP-COG IA (single stance phase), vertical ground reaction force (post-standardization), joint stiffness, and EMG. This impact on gait significantly differed among three dual tasks—texting > browsing > dialing—which confirms our hypotheses.

### 4.1. Walking While Using a Mobile Phone Induces Measurable Adjustment of Motor Pattern

Gait speed is an essential indicator for evaluating differences in dual-task walking, which could reflect the changes in gait [23,24]. Furthermore, other spatial–temporal parameters (step length, stride length, cadence, etc.) were influenced by gait speed. In our study, gait speed was significantly reduced when walking while using a mobile phone, which has been widely demonstrated [10,25].

Several studies have investigated the relationship between lower limbs and gait speed [26,27]. These alterations are consistent with a decrease in locomotor performance. In this study, the hip flexion angle decreased significantly during most of the gait cycle, which was consistent with the trend of hip torque and increased activation of the biceps femoris. This adaption caused inadequate flexion during the swing phase, decreasing the amplitude of the forward swing of the lower limb [8,24]. In addition, the angle of ankle plantar flexion decreased significantly during the swing phase, which was consistent with the increased activation of the gastrocnemius. It has been proven that foot contact area was positively associated with gait stability at the moment of foot strike [28]. While walking with mobile phone use, the subjects would adjust their posture actively to restore the initial position. This adjustment also reduced impact force during the single stance phase, which could be found in GRF.

The motion of COM and the relative position of COP of the supporting foot were among the most critical factors in assessing gait stability during level and stair walking. The COM-COP IA can characterize the whole-body position concerning the supporting foot [29]. Higher medial and posterior tilt angles were significantly associated with falls [21,29]. As the subjects have cushioned positively to keep a cautious gait, the posterior/anterior tilt angles decreased during the single stance phase. These alterations reflect that the range of body movement in the sagittal plane decreased while walking with mobile phone use, which was consistent with the trend of lower limb movement. There was a significant correlation between excessive joint stiffness and joint fatigue [10]. In addition, although no joint injury was found in the subjects during the experiment, the increase in ankle stiffness may reflect the higher risk of ankle fatigue, even injury.

### 4.2. The Reason for the Difference in Motor Performance Deterioration under Four Tasks

Based on the kinematic results, the impacts of the three tasks on gait are different; texting had the heaviest effect, followed by browsing, and finally dialing. The difficulty of the tasks might be one of the reasons, and the levels of interference in visual and cognitive demand might be the other reason. According to the hierarchical model, the interaction of higher- and lower-level control elements may affect task execution. The lower-level elements consist of well-practiced tasks (talking and walking), which could run simultaneously. On the contrary, combining lower and higher-level elements (texting and walking) requires sequential processing.

The impact of dialing was lower than the other two tasks; visual demand may be the most important reason. The visual factor was important in the motor gait [30,31,32,33]. Especially in our study, the subjects were required to step on the force platform, which added to their visual demand. When the subjects walked while browsing, their central vision was occupied, resulting in reduced gait speed, step length, joint angles, etc. This result has been proven by previous studies [31,32,33]. As the subjects lose their vision partly or even totally, they may be too scared of the dangers of their surroundings and choose a more cautious gait that could prevent injury.

Walking while using mobile phones increased the subjects’ cognitive workloads in many factors, such as attention, memory, executive control, etc. [1,17,34,35,36]. The impact of texting was more serious than browsing, and cognitive demand may be the key factor. Texting required subjects to “receive” information and “send” information while browsing only required subjects to “receive” without needing to “send” them to researchers. The results showed that texting significantly affects gait more than other tasks, consistent with previous studies [17,34,35]. The previous study has found that dialing may engage only low-level elements whereas walking while texting may require the execution of a high-level and low-level element [35].

### 4.3. The Underlying Mechanism of Motor Control Patterns Induced by Mobile Phone Tasks

Similar to previous results, walking with mobile phone use resulted in the subjects walking “smoothly” but more slowly [9]. This effect was more significant as the difficulty of tasks increased. However, this “stability” resulted from sacrificing the efficiency of movement. The biceps femoris and rectus femoris co-contract during the swing phase to stabilize the hip and reduce the range of motion, decreasing the gait speed. In addition, the gastrocnemius and tibialis anterior co-contract may also stabilize the ankle to increase the foot contact area. This co-contract has been proven to increase joint stiffness [37], which was consistent with the increase of hip and ankle stiffness in this study. Therefore, we suggest that this “hip–ankle strategy” during balance recovery was deliberate and tense, which may consume more unnecessary energy.

From the perspective of motor control, the deterioration of motor performance may be due to the overlap of the two tasks (walking and mobile phone use) at different processing stages, which results in a conflict at an information processing level. These results reflect the preference of the neuromechanical system to simplify control of motion in response to increased demands at the expense of slower motion. However, the essence of walking is to reach the destination quickly and accurately. The “stability” caused by the decrease in gait speed is contrary to the essence of walking. In this study, browsing, dialing, texting, and walking may conflict, as they all need planning and error correction. In reality, when people need to use a mobile phone while walking, they must have particular needs and, in the condition, ensuring the quality of these needs is of primary importance. Therefore, the main goal of this experiment was not movement control ability but keeping the accurate execution of the task. However, it has been proven that the accuracy of tasks would decrease if the gait speed were controlled [9].

This study combined balance control and joint injury indicators with kinematic/kinetic parameters to systematically analyze the effect of different mobile phone use tasks on gait and reveal the underlying mechanism. The comprehensive test tools support the multiple-perspectives analysis. In addition, our tasks were more related to daily life, which reflects reality better. The study had at least two limitations. Firstly, limited to the laboratory’s equipment, we did not test the upper limb, which could be further investigated in the future. Secondly, we did not control the gait speed and the accuracy of typing, which could prove the results better.

## 5. Conclusions

The overlap of walking and mobile phone use affects the gait significantly. The “hip–ankle strategy” may result in a “smooth” but slower gait, and this strategy was deliberate and tense. In addition, this adjustment also increases the stiffness of the hip and ankle, increasing the risk of fatigue. The difficulty of tasks due to the levels of interference in sensory and perceptual contribution and cognitive demand may cause the difference in dialing, browsing, and texting. Findings regarding this effect may prove the importance of simplifying the control of the movement. The effect may be more significant in individuals with worse cognitive/physical capabilities, further studies should quantify the potential risk of falls in similar conditions. Therefore, minimizing the physical alteration or visual distraction associated with cell phone manipulation may reduce the risk of falls.

## Data Availability

Not applicable.
